# Autism and social anxiety in children with sex chromosome trisomies: an observational study

**DOI:** 10.12688/wellcomeopenres.15095.2

**Published:** 2019-09-02

**Authors:** Alexander C. Wilson, Judith King, Dorothy V.M. Bishop

**Affiliations:** 1 Department of Experimental Psychology, University of Oxford, Oxford, OX2 6GG, UK; 2 Department of Psychiatry, University of Oxford, Oxford, UK

**Keywords:** Autism, social anxiety, trisomy X, Klinefelter syndrome, XYY syndrome, DAWBA, SRS, ascertainment bias

## Abstract

**Background**: Recent studies suggest that an extra sex chromosome increases the risk of both autism and social anxiety, but it unclear whether these risks are specific to particular karyotypes.

**Methods**: We considered diagnostic data from an online psychiatric assessment (DAWBA – The Development and Well-Being Assessment) and questionnaire responses completed by parents of children with 47,XXX (N = 29), 47,XXY (N = 28) and 47,XYY (N = 32) karyotypes. Analysis focused mainly on 54 children who were diagnosed prenatally or on the basis of other medical concerns in childhood (Low Bias subgroup), to minimise ascertainment bias.

**Results**: Children with symptoms of autism who fell short of meeting the Diagnostic and Statistical Manual of Mental Disorders (DSM)-IV criteria were coded as cases of Pervasive Developmental Disorder Not Otherwise Specified (PDDNOS). The odds ratio of autism or PDDNOS in the Low Bias group was computed relative to gender-specific population norms. This gave log odds ratio (95% confidence interval) of 5.56 (4.25 - 6.88) for XXX girls; 4.00 (2.66 - 5.33) for XXY boys; and 4.60 (3.46 - 5.74) for XYY boys. Despite this elevated risk, most children had no autistic features. A diagnosis of DSM-IV Social Phobia was rare, though, in line with prediction, all three Low Bias cases with this diagnosis had 47,XXY karyotype. All three trisomy groups showed increased risk of milder symptoms of social anxiety.

**Conclusions**: An increased risk of autism was found in girls with 47,XXX karyotype, as well as in boys with 47,XXY or 47,XYY. Symptoms of social anxiety were increased in all three karyotypes. There was wide variation in psychiatric status of children with the same karyotype, suggesting that an extra sex chromosome affects developmental stability in a non-specific way, with a diverse range of possible phenotypes.

## Introduction

Chromosome trisomies arise from an error of cell division during meiosis, so that either the egg or sperm contains two copies of the chromosome rather than one. When a trisomy affects one of the autosomes, this is often lethal, or causes severe physical and mental abnormalities. Trisomies of the sex chromosomes, however, have much milder effects, and often go undetected. In an analysis of 244,848 UK Biobank female participants, researchers discovered 110 cases of trisomy X, none of whom appeared aware of the trisomy ( Tuke *et al.*, 2017). Undiagnosed cases make study of the impact of sex chromosome trisomies difficult, because those cases who do come to attention may be atypical, with genetic testing being prompted by developmental abnormalities.

In the 1960s, several centres came together with the aim of evaluating the impact of sex chromosome trisomies in samples identified on newborn screening. The three kinds of trisomy - trisomy X (47,XXX), Klinefelter’s syndrome (47,XXY) and 47,XYY karotypes (henceforth referred to as XXX, XXY and XYY) were all found to be associated with neurodevelopmental problems, particularly affecting language and motor functions, though varying in profile and severity both within and between the three karyotypes ( Leggett *et al.*, 2010).

More recent studies, however, have noted an increased risk of autism in males: both those with Klinefelter’s syndrome and boys with an extra Y chromosome ( Bishop *et al.*, 2011; Cordeiro *et al.*, 2012; Joseph *et al.*, 2018; Ross *et al.*, 2012; van Rijn *et al.*, 2008), and in girls with trisomy X ( Wigby *et al.*, 2016). Furthermore, in all three trisomies, Bishop *et al.* (2011) found evidence of milder autistic-like communication problems affecting pragmatic as well as structural domains of communication, even after excluding those with a diagnosis of autism.

In one of the few studies to include girls and boys, Van Rijn and colleagues ( van Rijn *et al.*, 2014b) compared a sample of males and females (aged 9–18 years) with an extra X chromosome to a group of children with an autism diagnosis, using the Autism Diagnostic Interview - Revised (ADI-R) ( Le Couteur *et al.*, 1989). The trisomy samples included both prenatally identified and later-referred children but no differences were found between these subgroups. Furthermore, there were no differences between boys and girls with an extra X chromosome, with around 20% of children scoring above cutoff on the ADI-R in all three domains. Children were also given the Social Responsiveness Scale (SRS) ( Constantino, 2005).The rationale behind the SRS is that autism is not a qualitatively distinct condition, but is the extreme point on a continuum of impairment. The SRS was developed to quantify social and related impairments characteristic of autism in the broader population, and is scored so that the population mean is 50 (SD = 10), with high scores corresponding to impairment. Van Rijn *et al.* found that total SRS T-scores in the extra X group (XXX M = 63.8, SD = 31.7; XXY M = 68.7, SD = 31.7) were higher than those of a typically developing group (M = 26.3, SD = 16.3), but lower than an autism comparison group (M = 97.6, SD = 28.8). 44.2% of the extra X group scored above the threshold indicating clinically significant impairment (defined here as 65). This study, then, suggested that rates of autistic symptomatology are far higher in children with an extra X chromosome than was previously thought. However, results from the SRS do need to be interpreted cautiously. There is mounting evidence that while the SRS may be sensitive to autism, it is not very specific. A number of studies have shown that high scores on the SRS are not restricted to those with a diagnosis of autism, but are also reported for young people with a range of diagnoses, including conduct disorder, ADHD and anxiety, e.g., ( Bölte *et al.*, 2011; Cholemkery *et al.*, 2014; Moul *et al.*, 2015; Pine *et al.*, 2008; Settipani *et al.*, 2012; South *et al.*, 2017; Towbin *et al.*, 2005). In a sample of boys with XXY studied by Tartaglia *et al.* ( Tartaglia *et al.*, 2010), over 25% scored in the mild-to-moderate or severely impaired range in all domains of social responsiveness with the exception of social awareness, yet, in a subset of 20 children from this sample given a more detailed autism assessment, only one boy met diagnostic criteria for a pervasive developmental disorder.

Another intriguing finding from the study by van Rijn *et al.* (2014b) was that the extra X groups also showed high levels of social anxiety on all five subscales of the Social Anxiety Scale (SAS), with mean scores being higher than both the autism and the typically-developing group. The authors noted that this finding of increased social anxiety was consistent with earlier descriptive accounts of the impact of XXX and XXY karyotypes. Comorbidity between autism and social anxiety is common in children with typical chromosome complements ( Spain *et al.*, 2018), but the particularly high rates of social anxiety in the children with trisomies suggests that this might be a separate condition, not just secondary to autism. As noted by van Rijn *et al.* (2014b), this suggests that ‘children with an extra X chromosome differ from children with ASD in an important aspect, which is the ability to reflect on their own social functioning and to develop a related concern about social rejection, i.e., the opinions, thoughts and expressions of others.’ (p. 317)

Most of the focus of studies of children with sex chromosome trisomies using the SRS has been on the impact of an additional X chromosome, Cordeiro and colleagues (2012) compared boys with XXY and XYY chromosome complements, and found that the XXY group had significantly less impairment than the XYY group on the total SRS T-score (XXY M = 62.0, SD = 15.4; XYY M = 72.8, SD = 15.8). There was, however, evidence of ascertainment bias in this study, with prenatally diagnosed children (XXY M = 58.6, SD = 14.2; XYY = 63.5, SD = 15.6) having lower total SRS T-scores than postnatally diagnosed children (XXY M = 66.3, SD = 16.1; XYY M = 77.3, SD = 14.1). 47.1% of all the XXY children and 85% of the XYY children scored above the threshold for social impairment (defined as 60). Scores on individual SRS subscales were very similar to total scores, with significantly more impairment for XYY children compared to XXY children on all subscales, except Social Motivation, on which they scored the same; this scale represented a relative strength for the XYY group in terms of their overall profile. For the XXY group, means were above the 60 cut-off on all subscales except Social Awareness, which represented a relative strength for this group. Interestingly, these patterns for XXY boys to score relatively better on Social Awareness than other subscales and for XYY boys to score relatively better on Social Motivation than other subscales have been replicated in other studies (XXY: ( Tartaglia *et al.*, 2010) XYY: ( Ross *et al.*, 2015)). This pattern has suggested that there may be ‘characteristic profiles’ of social difficulties in children with SCTs. This is reminiscent of earlier work that suggested that Klinefelter’s Syndrome was associated with a particular personality style, characterised as shy, sensitive and passive ( Herlihy *et al.*, 2011). More recently, Joseph *et al.* (2018) found that elevated SRS scores were characteristic of males with XYY karyotype, especially those identified postnatally, but the specificity of the SRS was poor, with high scores associated with a range of psychiatric symptomatology.

Taken together, these studies indicate that an increased likelihood of a clinical-range SRS score is seen in children who have an extra X or Y chromosome, though the pattern of results suggests there may be a different profile depending on whether there is an additional X or Y chromosome. Studies of children who do not have trisomies indicate a need for caution in interpreting high SRS scores, as the specificity of this instrument is poor. In particular, children and young people with social anxiety may score within the clinically significant range of the SRS. Disentangling the relationship between autism and social anxiety is not straightforward because anxiety-related social behaviours may look like autism, but have a different underlying basis ( Tyson & Cruess, 2012).

The goal of the current study was to evaluate profiles of social impairment and autistic features in children with an extra sex chromosome using information from an online diagnostic instrument (The Development and Well-Being Assessment - DAWBA) and parental questionnaires, including the SRS.

Specific predictions were:

1.   In line with our previous study ( Bishop *et al.*, 2011), we expected to find an increased rate of autistic features in all three trisomies, but with confirmed autism diagnoses elevated only in boys with XXY or XYY.

2.   Social anxiety symptoms were expected to be higher in children with an extra X chromosome (XXY and XXX) than in boys with an extra Y.

3.   We anticipated that ascertainment bias will affect results, and levels of social and psychiatric impairment will be higher in children identified in the course of investigation for developmental disorders, than in other children with sex chromosome trisomies.

4.   Finally, extending the work by Joseph *et al.* (2018), we expected that children with all three types of trisomy will have elevated scores on the SRS, but this will not necessarily be indicative of autism.

## Methods

### Participants

**Figure 1.**
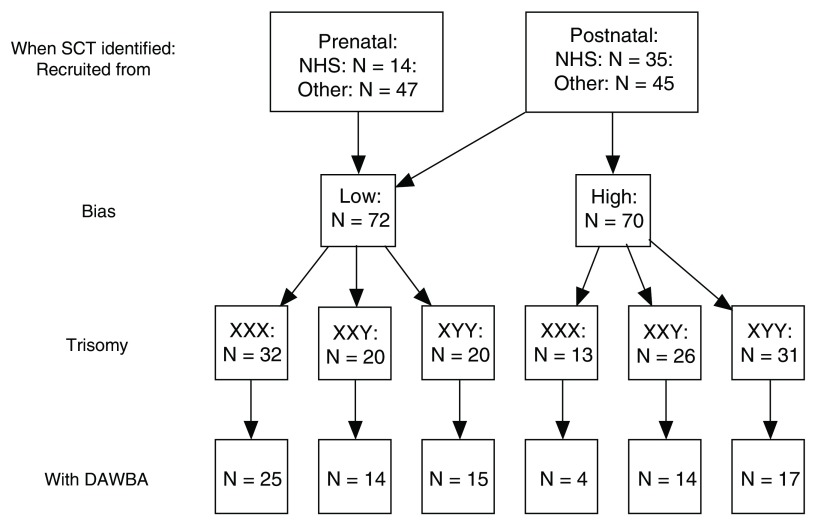
Flowchart showing numbers of children with each karyotype in the study, in relation to whether prenatally or postnatally diagnosed, whether high ascertainment bias, and whether DAWBA was completed. SCT = Sex chromosome trisomy; NHS = National Health Service; DAWBA = Development and Well-Being Assessment.

The full sample included 89 children (29 with XXX, 28 with XXY and 32 with XYY) whose parents had completed the online Development and Wellbeing Assessment (DAWBA, see below). As shown in Figure 1, these came from a larger sample of 142 children aged from 5 to 16 years who participated in a study of language and laterality in sex chromosome trisomies; genetic, laterality and language characteristics of these children have been reported in previous papers ( Bishop *et al.*, 2018; Newbury *et al.*, 2018; Wilson & Bishop, 2018). Figure 1 shows recruitment sources as via National Health Service Clinical Genetics centres, or Other (via two support groups: Unique: the Rare Chromosome Support Group, and the Klinefelter Syndrome Association, or from self-referral via social media). A criterion for inclusion was that the child was aware of their trisomy status. Figure 1 distinguishes between cases where the trisomy was discovered prenatally or in the course of medical investigations unrelated to neuropsychiatric problems (e.g., problems affecting hormonal or muscular-skeletal systems), and those discovered in childhood during investigations for behavioural or neurodevelopmental problems. The latter group, referred to as the ‘High Bias’ subgroup, are likely to have a high rate of disorder that is not representative of that karyotype. Accordingly, most of the analysis reported here excludes these cases and focuses on the ‘Low Bias’ subgroup. An unexpected feature of the sample was that the majority of girls with trisomy X were in the Low Bias subgroup, whereas the majority of boys with XXY or XYY were in the High Bias subgroup, suggesting that these karyotypes were more likely to be associated with neurodevelopmental problems. The Low Bias and High Bias subgroups were comparable in age: mean (SD) for Low Bias was 10.60 (3.70) years, and for High Bias 10.80 (4.0) years.

One advantage of using DAWBA is that there are comprehensive epidemiological British data for boys and girls available using this instrument ( Meltzer *et al.*, 2000). In order to compare rates of psychiatric problems with population prevalence figures from Meltzer *et al.* the sample was subdivided into a younger (age 5–10 years) and older (age 11–16 years) age group. Numbers in these two age bands were roughly equal for all karyotype groups: Low Bias: XXX (14 younger, 11 older); XXY (9 younger, 5 older), XYY (9 younger, 6 older); High Bias: XXX (2 younger, 2 older); XXY (6 younger, 8 older), XYY (10 younger, 7 older).

23 children had taken part in the previous study by Bishop *et al.* (2011). Information about autism in that study was available only from parental report: two of the children (8.7%) had been reported by their parents to have a diagnosis of autism. No information on social anxiety had been collected in the 2011 study.

In a previous report based on the current sample ( Bishop *et al.*, 2018), we noted that the likelihood of parents completing checklists for this study was lower if the child had significant language problems. This prompted us to compare children whose parents did and did not complete the DAWBA, using a vocabulary T-score (see below) as an indicator of language level. A t-test revealed that, as before with checklist completion, parents who completed the DAWBA had children with milder language problems: mean vocabulary score for those who completed DAWBA was 38.9 (SD = 11.62) versus 32.3 (SD = 8.60) for the remainder, t (119.7) = 3.71, 95% CI for difference in means = 3.12 to 10.28.

## Material

### Psychiatric evaluation

One or both parents were asked to complete the online Development and Wellbeing Assessment (DAWBA) ( Goodman *et al.*, 2000) in their own time; 88 families of children with a SCT (one with two affected children) complied with this request.

The DAWBA includes a range of questions relating to DSM-IV and ICD-10 diagnostic criteria, risk factors, and impact on the family, which are supplemented by optional free text.

The DAWBA software codes the items relevant to different diagnoses into bands that indicate the likelihood of the child meeting criteria for a range of psychiatric diagnoses ( Goodman *et al.*, 2011), with the final diagnosis being made by a trained rater who assimilates all the quantitative and qualitative information. DAWBA has been used to obtain prevalence estimates for psychiatric disorders for a representative sample of over 10,000 children in Great Britain ( Meltzer *et al.*, 2000). Validity of DAWBA for diagnosis of autism has been shown to be acceptable ( McEwen *et al.*, 2016).

All interviews with SCT cases were coded against DSM-IV criteria by the first author (AW); 30 interviews were recoded by a second independent rater (JK), with the remainder recoded by the last author (DB). The rater doing the coding was blind to the trisomy status of the child, although occasionally this was mentioned by parents in free text comments. Cases of disagreement were resolved by the last author (DB), who gave a final consensus rating that was used here.

To address our questions of interest, we focused on DSM-IV diagnoses of Autism Spectrum Disorder (ASD) and Social Anxiety Disorder. ASD was coded as absent (0) or present (2), with a code of 1 given for significant autism symptoms that fell short of diagnostic criteria. Most of the latter group, which we refer to as the Pervasive Developmental Disorder Not Otherwise Specified (PDDNOS) group, showed evidence of social and communicative difficulties, but did not have sufficient repetitive behaviours and restricted interests to merit an autism diagnosis. There was agreement between raters on autism coding for 83.10% of cases, disagreement by one point (ratings of 0/1 or 1/2) in 12.40%, and major disagreement (ratings of 0/2) in 4.50%. There were 24 children whose parents reported an existing diagnosis of autism, and all of these were identified as definite autism by DAWBA. Meltzer *et al.* (2000) provided prevalence figures for a broader category of PDD (pervasive developmental disorder) so we combined autism and PDDNOS when comparing diagnoses with their sample.

The criteria for DSM-IV Social Phobia (now more commonly known as Social Anxiety Disorder) include a marked and persistent fear of one or more social situations where the individual may be subject to scrutiny by others. For a diagnosis of Social Phobia the symptoms must interfere significantly with everyday life. In children, the anxiety must occur in peer settings, not just with adults, must be disproportionate to the situation, and not be better explained by another disorder such as ASD. Social Phobia is differentiated from Separation Anxiety and General Anxiety Disorder by the situational specificity of the anxiety. Social Phobia was coded as present or absent, with 93.30%, agreement between raters.

### The Social Responsiveness Scale (SRS)

The SRS is a 70-item scale designed to measure autism-related impairments as quantitative traits. It is composed of five subscales: social cognition, social awareness, social motivation, social communication and autistic features. Scores on each subscale, and on the total scale, were transformed to T-scores (based on the test manual) with mean 50 and SD of 10, with impairment represented as a positive score.

### Vocabulary measure used to compare those with and without DAWBA scores

The Vocabulary subtest from the Wechsler Abbreviated Scale of Intelligence ( Wechsler, 1999) administered to children as part of the language test battery ( Bishop *et al.*, 2018) was used to check whether the families who completed DAWBA differed from others (see above).

### Additional measures not included in this analysis

Children in this study were given additional language and cognitive assessments, which are described fully in the companion paper by Bishop *et al.* (2018), and an assessment of cerebral and manual laterality, as documented by Wilson & Bishop (2018). DNA from saliva samples was analysed, as described by Newbury *et al.* (2018).

### Procedure

Ethical approval was obtained for the study in 2011 from the Berkshire NHS Research Ethics Committee (reference 11/SC/0096), and data collection started in August of that year, finishing in October 2016. Families who had expressed interest in the study were interviewed by telephone to assess whether the child met inclusion criteria, and if so, a parent was invited to complete a consent form. Those who gave consent were also asked to view a video (available from YouTube) about the study with their child to ensure the family understood what was required before asking the child’s assent to take part. For those who agreed, an appointment was made to see the child at home or at school, depending on parental preference. Families were widely dispersed around the UK, including Northern Ireland, Scotland, Wales and England. Parents were provided with passwords and a personal link to access the secure DAWBA website. They were asked to complete the DAWBA in their own time, and if they wished could spread the task over several days. The SRS was either completed by the parent while the child was doing language assessments, or was mailed to the parent and returned by post.

### Data analysis and power calculations

Study data were analysed using R software, version 3.5.0 ( R Core Team, 2016), with the main database managed using REDCap electronic data capture tools hosted at the University of Oxford ( Harris *et al.*, 2009). The original sample size had been determined to give adequate power for genetic analyses reported by Newbury *et al.* (2018), rather than with phenotypic analyses in mind, and so we conducted additional analyses to consider the power for the current analyses on the reduced sample with DAWBA data.

 For comparing rates of disorder in the Low Bias group with population prevalence figures from Meltzer *et al.* (2000), we used the log odds ratio. For this comparison, the smallest samples used here (N = 14) is sufficiently powered to detect an increase of prevalence of 20% or more, albeit with a large confidence interval. For instance, if the general population prevalence of disorder is 5%, and 3/14 of a trisomy group has disorder, then the log odds ratio is log((3/11)/5/95))= 1.64. The standard error of the log odds ratio is the square root of the sum of the reciprocals of the four frequencies ( Bland & Altman, 2000). Since the sample size for Meltzer *et al.* is over 5000 for both boys and girls, then for our example, the standard error would be approximately sqrt(1/250+1/4750+1/3+1/11) = 0.65, so the 95% confidence interval for the log odds ratio covers 0.36 to 2.91.

For quantitative comparisons between the three Low Bias trisomy groups, we used nonparametric Kruskal Wallis tests. A rough estimate of power can be obtained using the *pwr.anova.test* function in R ( Champely, 2018). This gives a value of f = 0.25, corresponding to a medium effect size in Cohen’s (1988) terminology with 80% power and alpha of 0.05. However, actual power is likely to be lower than this because of the use of unequal group sizes and non-normal measures. We made a further set of comparisons between the Low Bias and High Bias groups, with all karyotypes combined. Using the *pwr.t2n.test* function for computing power of t-test with unequal sizes, gives 80% power to detect a Cohen’s d of 0.61 with alpha of 0.05.

Thus for karyotype comparisons in the Low Bias trisomy cases, and between the combined Low Bias vs High Bias groups, our sample is powered to detect only relatively large effects. More subtle effects will require larger samples, which are unlikely to be feasible for a single research centre. This emphasises the need for raw data to be available so that future researchers will be able to combine information across studies.

## Results

Our research questions focused on the rates of autism and Social Phobia in XXX, XXY and XYY karyotypes. Because we predicted that these rates would be influenced by ascertainment bias, the data were analysed separately for the Low Bias and High Bias subgroups. Table 1 shows the numbers of children with DSM-IV diagnoses of autism, PDDNOS or Social Phobia on the Development and Wellbeing Assessment (DAWBA).

**Table 1.** Numbers (percentages) by karyotype group for DAWBA diagnosis of autism, PDDNOS and Social Phobia. (PDDNOS indicates with autism symptoms but falling short of DSM-IV criteria). DAWBA: Development and Well-Being Assessment, DSM-IV: Diagnostic and Statistical Manual of Mental Disorders IV, PDDNOS: Pervasive Developmental Disorder Not Otherwise Specified.

		Low Bias			High Bias	
Diagnosis	XXX	XXY	XYY	XXX	XXY	XYY
N	25	14	15	4	14	17
Neither	20 (80%)	9 (64.3%)	10 (66.7%)	2 (50%)	7 (50%)	4 (23.5%)
PDDNOS	3 (12%)	1 (7.1%)	1 (6.7%)	1 (25%)	1 (7.1%)	3 (17.6%)
Autism	2 (8%)	1 (7.1%)	4 (26.7%)	1 (25%)	2 (14.3%)	9 (52.9%)
Social Phobia only	0 (0%)	2 (14.3%)	0 (0%)	0 (0%)	0 (0%)	0 (0%)
Social Phobia+PDDNOS	0 (0%)	1 (7.1%)	0 (0%)	0 (0%)	0 (0%)	0 (0%)
Social Phobia+Autism	0 (0%)	0 (0%)	0 (0%)	0 (0%)	4 (28.6%)	1 (5.9%)

Our first categories of prediction was that there would be a high prevalence of autism or PDDNOS specifically in boys with XXY or XYY. Meltzer *et al.* (2000) reported prevalence rates for ‘Pervasive Developmental Disorder’ (encompassing autism and PDDNOS) of 0.5% for boys and 0.1% for girls. Compared with these figures, there are elevated rates in all three trisomies. We consider here just the Low Bias groups: the log odds ratio (with 95% confidence interval) is 5.56 (4.25 - 6.88) for XXX girls; 4.00 (2.66 - 5.33) for XXY boys; and 4.60 (3.46 - 5.74) for XYY boys. One of the affected girls had been in the original 2011 study, but had not been identified with ASD at that time. Our prediction of an increased prevalence of ASD was confirmed, but this extended to girls with XXX as well as the two groups of boys. The prevalence rates reported by Meltzer *et al.* (2000) are lower than subsequent estimates of autism prevalence in the UK ( Baird *et al.*, 2006), but we found that even if we doubled the prevalence rates used to compute odds ratios, the frequencies were still well above expected levels. Nevertheless, those with an autism diagnosis were in the minority in all three trisomies.

Our second prediction was that there would be a raised prevalence of Social Phobia in those with an extra X chromosome. As shown in Table 1, there were three children with this diagnosis in the XXY-Low Bias group and four in the XXY-High Bias group, as well as one child in the XYY-High Bias group. All but two of these children had additional diagnoses of autism or PDDNOS. Although the numbers of boys with XXY studied here is very small, the finding that 21% in the Low Bias group and 28% in the High Bias group met diagnostic criteria for Social Phobia is well above the prevalence in the general population (log odds ratio for Low Bias group = 4.21, 95% CI = 2.87-5.56). It may seem plausible that Social Phobia would be a condition that develops with age, as children’s ability to reflect on the reactions of others develop and social demands on them increase. However, the effect of age was small in the epidemiological sample of Meltzer *et al.* (2000), with prevalence of Social Phobia at 0.4% in young boys, 0.2% in young girls, 0.3% in older boys and 0.4% in older girls. There was no obvious association with age in the trisomy sample: the three XXY boys with Social Phobia in the Low Bias group were aged 6, 10 and 14 years.

Since the initial version of this paper was published, a new government report has been published on Mental Health of Children and Young People, 2017 (MHCYP), based on a representative sample of children and young people in England ( NHS Digital, 2018). Survey methods were similar to those used by Meltzer *et al.* (2000), with diagnoses based on the DAWBA. Diagnostic data are available for 1819 boys and 1778 girls in the age range 5 to 10 years, and 1553 boys and 1568 girls aged 11 to 16 years. In this sample, rates of autism/PDDNOS were higher than in the previous population sample: 2.5% for boys and 0.4% for girls in the age range 5 to 10 years, and 1.8% for boys and 0.7% for girls in the age range 11 to 16 years. Rates of autism/PDDNOS and social phobia were compared against these new norms, using a prevalence rate weighted to take into account proportions of children in the younger and older age bands. Results were generally unchanged, except for autism/PDDNOS in the Low Bias group with XXY. Relative to the recent norms, the log odds ratio for the Low Bias group with XXY was no longer raised relative to population levels: 1.22 (95% CI from -0.82 to 3.27). Although the MHCYP data also show an increase in rates of social phobia in older children, the elevated rates in XXY groups remained well above expected levels.

DAWBA categorical diagnoses only detect more severe levels of impairment associated with burden to families. We therefore also examined symptoms reflected in the DAWBA banding for Social Phobia ( Goodman *et al.*, 2011), an ordinal scale computed from the profile of social anxiety symptoms. Table 2 shows the distribution of DAWBA band scores. In the British Child and Adolescent Mental Health Surveys (B-CAMHS) sample, 96% of cases fell in band 0 or 1. It is evident by inspection that subclinical levels of symptoms are relatively high in all three types of trisomy. For the Low Bias group, the log odds ratios (95% CI) for scoring in band 2 or above, relative to the B-CAMHS sample is 7.60 (6.20 - 9) for XXX girls, 7.60 (6 - 9.10) for XXY boys, and 6.90 (5.30 - 8.60) for XYY boys. Although the trend is for a higher rate of problems in those with an extra X chromosome, the difference in rates of scores of band 2 or above by karyotype was not statistically reliable; Kruskal-Wallis chi-squared = 1.43, df = 2, p = 0.49.

**Table 2.** Numbers (percentages) by karyotype group in DAWBA bands for Social Phobia, in relation to data from follow-up of the British Child and Adolescent Mental Health Surveys (B-CAMHS; Goodman *et al.*, 2011). DAWBA: Development and Well-Being Assessment.

Band	B-CAMHS	XXX-low	XXY-low	XYY-low	XXX-high	XXY-high	XYY-high
0	6514 (83.8%)	8 (32%)	6 (42.9%)	7 (50%)	2 (50%)	5 (35.7%)	6 (35.3%)
1	996 (12.8%)	6 (24%)	2 (14.3%)	3 (21.4%)	0 (0%)	3 (21.4%)	7 (41.2%)
2	208 (2.7%)	10 (40%)	3 (21.4%)	4 (28.6%)	1 (25%)	2 (14.3%)	2 (11.8%)
3	41 (0.5%)	0 (0%)	1 (7.1%)	0 (0%)	1 (25%)	0 (0%)	1 (5.9%)
4	18 (0.2%)	1 (4%)	2 (14.3%)	0 (0%)	0 (0%)	4 (28.6%)	1 (5.9%)

**Figure 2.**
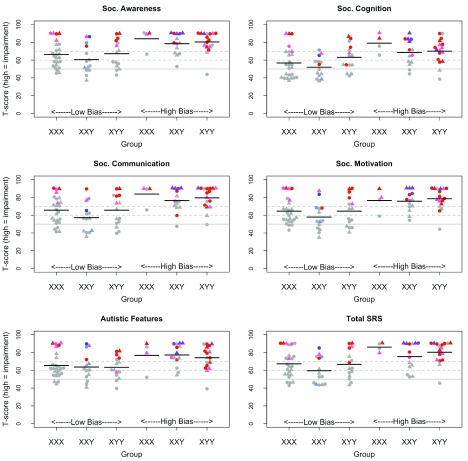
Beeswarm plots: Social Responsiveness Scale (SRS) scales and total T-score for the three trisomy groups, subdivided by bias group. The horizontal lines show population mean (50, solid line), plus cutoffs of 60 and 70 (dotted lines), corresponding to mild and more severe problems. Symbol depicts age band (circle 5–10 yr, triangle 11–16 yr. Colour depicts diagnosis: grey = none; light blue = Social Phobia; dark blue = Social Phobia+PDDNOS; pink = PDDNOS; red = autism; purple = Social Phobia+autism).

Figure 2 shows the T-scores on the SRS. SRS T-scores are scaled with mean of 50 and SD of 10, where a high score indicates impairment. The figure shows individual data points as well as means on the SRS scales, with children meeting diagnostic criteria for autism and/or Social Phobia colour-coded.

It is evident from Figure 2 that, while the overall rate of impairment is raised relative to the general population, many children with sex chromosome trisomies score within normal limits. We had planned to use Multivariate Analysis of Variance (MANOVA) to compare groups on the SDQ and SRS, but assumptions of multivariate normality were not met, and so nonparametric Kruskal-Wallis tests were used instead. A randomisation test with 10,000 iterations was used to estimate the range of chi square values that would be obtained under the null hypothesis, where group identity was assigned at random. A series of analyses was conducted to address the specific questions raised in the Introduction.

**Figure 3.**
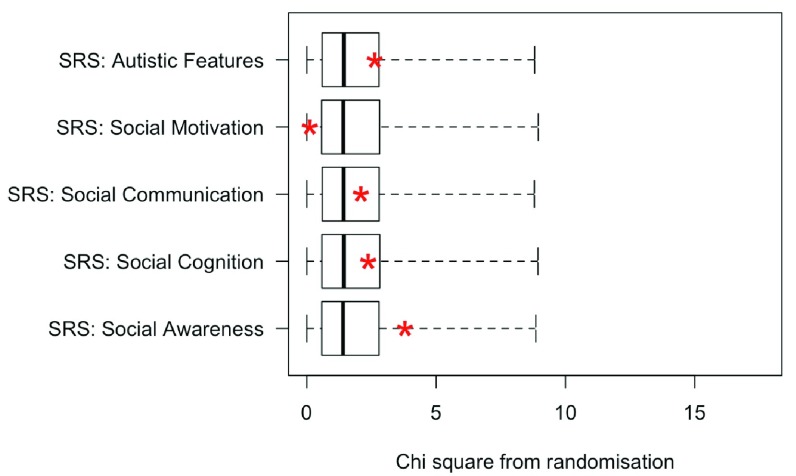
Plot of chi square values (red asterisks) obtained on Kruskal-Wallis test comparing the three Low Bias sex chromosome trisomy groups relative to the range of chi square values obtained in 10,000 iterations of randomisation test (i.e., when same analysis conducted with group status randomised). The box shows the interquartile range, and the upper fin of the boxplot corresponds to the 99th centile for randomised values.

**Test of hypotheses 1–2** (Low Bias group only). The first two hypotheses were that there would be chromosome-specific differences in profiles, with autistic features more prominent in boys with an extra Y chromosome, and other types of social impairment in boys and girls with an additional X chromosome. To reduce the effects of ascertainment bias, these predictions were tested using data from the Low Bias groups only. Scores for the three karyotypes were compared on the five SRS scales. Results are summarised in Figure 3, which shows the obtained chi square values in relation to those from the randomised data, with the upper fin of each boxplot corresponding to the 99th centile. Given the small sample sizes, and wide variation within each karyotype, the power is low to detect subtle effects.

**Figure 4.**
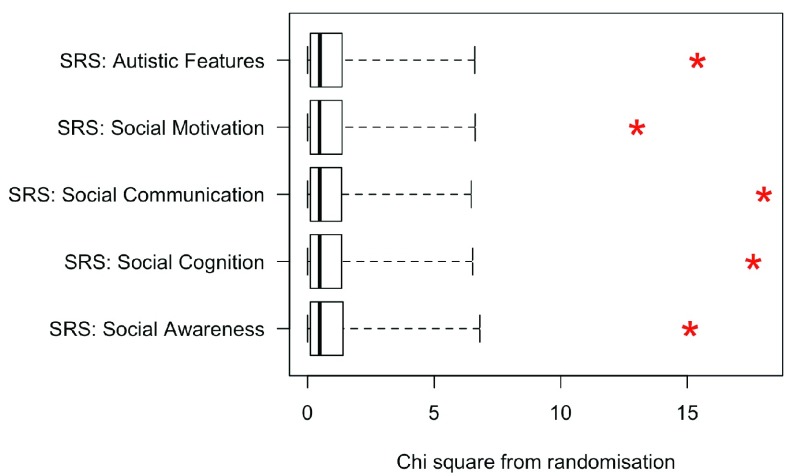
Plot of chi square values (red asterisks) obtained on Kruskal-Wallis test comparing Low Bias vs High Bias sex chromosome trisomy cases (all trisomies combined), relative to the range of chi square values obtained in 10,000 iterations of randomisation test (i.e., when same analysis conducted with group status randomised). The upper fin of the boxplot corresponds to the 99th centile for randomised values.

**Test of hypothesis 3**. (Low Bias vs High Bias trisomy groups) Hypothesis 3 predicts that children in the High Bias group will have more evidence of impairment than those in the Low Bias group. Again, multiple Kruskal-Wallis tests were used to test this prediction, this time with the three karyotypes combined. Results are shown in Figure 4. This analysis confirms the substantial differences between the Low Bias and High Bias groups, who differ markedly on all the scales.

**Test of hypothesis 4**. Hypothesis 4 proposed that elevated scores on the SRS would be seen in children with sex chromosome trisomies, but would not necessarily be indicative of autism. As shown in Figure 2, the odds of having a score outside the normal range on the SRS scales is elevated in all three trisomies, especially for children from the High Bias Group. The last panel of Figure 2 shows T-scores on the SRS total for children with sex chromosome trisomies in relation to DAWBA diagnoses of autism, PDDNOS and Social Phobia. These data show that all but one child with a diagnosis of autism or PDDNOS score above the ‘Severe’ cutoff of 70, but most children in the ‘Mild’ range of 60–70 do not have a diagnosis of either autism or PDDNOS.

A cutoff total score of 75 has been reported as giving sensitivity and specificity of 0.85 and 0.75 for autism diagnosis in a population of mixed cases of autism and other developmental disorders ( Bölte *et al.*, 2011). In the current sample, if we group together the autism and PDDNOS cases, then with this cutoff, we obtain 45 true negatives (non-autistic < 75), 9 false positives (non-autistic > 74), 1 false negative (autistic < 75), and 34 true positive (autistic > 74), which gives sensitivity of 0.97 and specificity of 0.83. If, however, we compute predictive power for diagnosis of autism, and treat above-cutoff PDDNOS cases as false positives, sensitivity remains high at 0.96 but specificity declines to 0.69. In general, our results are consistent with those of Bölte *et al.* (2011), in showing that a cutoff of 75 is effective at identifying children with autistic features, though many of those scoring in this range would not meet full DSMIV criteria, but rather correspond to cases of PDDNOS. Provided we adopt this more stringent cutoff, our data are reassuring in suggesting that the SRS does not identify a high proportion of non-autistic children.

## Discussion

Before discussing the specific results found here, it is important to consider potential biases in the sample of children with sex chromosome trisomies. We distinguished a High Bias group who were identified during investigation for neurodevelopmental or behavioural problems; these cases may be useful in revealing genetic or other factors that may be associated with variable outcomes, but they will inevitably over-estimate prevalence of problems. Our Low Bias group is more appropriate for establishing risk of various outcomes, but it is important to note that there still will be some bias in those identified on prenatal screening: mothers will be older than average, and will have elected to continue with the pregnancy. Because the prenatally-diagnosed cases were identified via National Health Services Clinical Genetics departments, they are less likely than those from US samples to be biased to more affluent families. However, another bias that is impossible to control is determined by who volunteers to take part in the research. In this study, children could be included only if they knew about their chromosome status; our prior research has shown that parents are more likely to disclose this information when the child is experiencing difficulties ( Gratton *et al.*, 2016). Children were also required to assent to take part in the study, after viewing a video that provided information about what was involved. It is plausible that this would deter children with significant social anxiety. In addition, only 89 of 142 (62%) of participating families completed the DAWBA, and our analysis showed that the child’s language level was a factor affecting this, with parents of more mildly affected children being more likely to complete the interview. Factors such as these will inevitably affect who takes part in any study of children with genetic conditions - even the original newborn screening studies - as one cannot compel people to participate. The best we can do in this situation is to be transparent about sources of bias, and consider how far results are consistent across different studies where specific influences on who volunteers may assume different importance. Bearing this in mind, we turn to consider the specific results obtained here, which lead to six broad conclusions.

### 1. Sex chromosome trisomy as risk factor for autism

The current results add to those of previous research ( Cordeiro *et al.*, 2012; Joseph *et al.*, 2018; Ross *et al.*, 2012; van Rijn *et al.*, 2014a; van Rijn *et al.*, 2008) in indicating that the rate of autism is increased in children with an extra sex chromosome. On the basis of previous results by Bishop *et al.* (2011), we predicted that elevated rates of autism would be found only in boys with XXY and XYY. However, our results were consistent with the more recent studies of van Rijn *et al.* (2014a) and Wigby *et al.* (2016), who found increased levels of autism in girls with trisomy X. In the current sample, two of 25 girls with XXX in the Low Bias group, as well as one of four girls in the High Bias group met DSM-IV criteria for autism. Furthermore, if we adopt the broader definition of PDDNOS, which includes cases with significant autism symptoms but falling short of autism (usually because of insufficient evidence of repetitive behaviours) then we can compare rates with the gender-specific population norms for PDD of Meltzer *et al.* (2000). Although those with autism or PDDNOS were a minority of children, and the sample was too small to give a confident estimate of prevalence, these results show that the increased prevalence of autism applies to all three trisomies. Note that a minority of children in the current study had taken part in the earlier study by Bishop *et al.* (2011), and all of those with autism in the current sample were new cases.

### 2. Sex chromosome trisomy as risk factor for social anxiety disorder

Very few children in this study met diagnostic criteria for DSM-IV Social Phobia: all but one of those who did were cases of XXY. Only two children (both from the Low Bias XXY group, N = 14) had Social Phobia in the absence of any autistic features. While this represents a substantial increase over expected values (relative to Meltzer *et al.*’s epidemiological prevalence of 0.4% in boys), numbers are too small for reliable estimates. When we considered milder symptoms of social anxiety, using DAWBA bands, a rather different picture emerged, with 11/25 (44%) of XXX girls, 6/14 (43%) of XXY boys and 3/13 (23%) of XYY boys being placed in band 2 or over, compared with a prevalence of 3.4% in the B-CAMHS epidemiological sample. Thus all three types of sex chromosome trisomy confer a risk of increased social anxiety, though this generally falls short of meeting diagnostic criteria for Social Phobia.

Five boys from the High Bias group (four with XXY and one with XYY) received a dual diagnosis of both Autism and Social Phobia. As noted by Raznahan (2019), these conditions can be hard to distinguish phenomenologically. The diagnostic criteria for Social Phobia include the statement that the anxiety should not be better explained by another disorder such as autism, requiring the clinician to be able to rate just how much social anxiety is explicable in those terms. In effect, one is attempting to determine whether avoidance of social situations is because of concerns about social judgements of others, or because poor social understanding makes social situations anxiety-provoking because they are unpredictable. DAWBA respondents were encouraged to add free text comments to supplement the response to multiple choice questions, and this could help make the differentiation, but they varied in the extent to which they did this. The authors took all information into account when converting DAWBA data to diagnoses, and added comments to explain their decisions, but clearly found this differentiation hard to make in some cases where there was dual diagnosis. For instance, for one boy, the rater speculated whether the social difficulties might be secondary to autism, stating: “High level of upset, avoidance and interference with normal routine caused by specific social fears. Present even with key adults around. The criteria stipulate that the symptoms should not be better explained by ASD diagnosis, and the fears seem to exceed the reduced social-emotional reciprocity, etc., of ASD. Also, note the ASD social deficits are not too severe, as indicated by the ASD form, and child does have friends and is not victimised.” In a contrasting case, again with dual diagnosis, the rater considered whether apparent autistic symptomatology might be a consequence of social anxiety. This child had withdrawn from social contact because he was self-conscious about his appearance, and the rater noted: “Parent repeats issues with child withdrawing, locking self in room, involvement of CAMHS. Very anxious out of the house. Not clear how much of ASD symptomology is secondary to social anxiety. Reports of child's anxiety eating in front of others, being spoken about, etc., suggests fears are sufficiently specific in addition to general anxieties for social anxiety diagnosis under DSM.”

A final point is that, as noted above, a child with severe Social Phobia is unlikely to have agreed to participate in the study, as this involved an individual assessment with an unfamiliar adult. Accordingly, we may have underestimated the frequency of this condition.

### 3. Many children show no signs of either autism or social anxiety

We used parental responses to the SRS to capture mild, subclinical aspects of psychiatric problems, and it did reveal elevated levels of impairment, even when children with diagnoses of autism/PDDNOS or Social Phobia were excluded. However, the SRS also confirmed the wide range of outcomes in all three trisomies, with around half the children in the Low Bias group scoring within the normal range. In this regard, our results are compatible with those of a recent study by Freilinger *et al.* (2018), who studied behavioural and psychological features in a sample of 72 girls and women with trisomy X, most of whom were diagnosed prenatally. They found that around half of them did not have behavioural problems.

### 4. The effects of ascertainment bias are substantial

As anticipated, children from the High Bias group had more severe problems than those from the Low Bias group. Although most studies that have looked at this question have found similar patterns, this is not always the case (cf. ( Van Rijn *et al.*, 2014a)). The method for dividing cases into Low Bias and High Bias subgroups may affect results: if the latter group is defined purely in terms of late diagnosis, then it may include some whose trisomy was discovered in the course of medical investigations, rather than, as in our sample, being restricted to those where behavioural or cognitive impairments had prompted genetic investigation.

### 5. More similarities than differences in the impact of an extra X or extra Y chromosome

The notion that supplementary X and Y chromosomes might have different effects is attractive as well as plausible ( Green *et al.*, 2019; Skuse, 2018), particularly since it has potential to throw light on sex differences in psychiatric disorders in general. However, data from the current study indicate that insofar as such effects occur, they can be swamped by other influences, leading to a highly heterogeneous picture.

We should not dismiss the idea that there could be complementary effects (more social anxiety with an extra X and more autism with an extra Y) on the basis of data from a single study. Our sample sizes were small, especially when attention was restricted to the Low Bias group, so we lacked statistical power to detect any but large associations. Interpretation is also complicated by changing prevalence rates for autism in the general population. The rate of autism/PDDNOS in boys with XXY was no longer outside population limits when compared to recent data from a 2017 population survey.

A further complication is that the proportions of children in the Low Bias and High Bias groups differed across trisomies, with relatively few girls in the High Bias group. To be included in the High Bias group, children had to present with developmental difficulties. Thus the relatively low proportion of girls with trisomy X in the High Bias group, compared to XXY and XYY might indicate that the social and behavioural consequences of a supernumerary sex chromosome are usually less severe in girls than in boys.

Insofar as there were trends in the data, they agreed with the prediction of higher levels of social anxiety in boys and girls with an extra X chromosome, and higher levels of autism and reduced prosocial behaviour in boys with an extra Y. But we can conclude that, insofar as such effects occur, risks for specific disorders are probabilistic rather than deterministic. A simple model that treats social anxiety and lack of social awareness as points at either end of a single dimension is also complicated by the finding of cases who met diagnostic criteria for both Social Phobia and autism/PDDNOS.

### 6. Variability in outcomes suggests that an extra sex chromosome creates general developmental instability

The more we discover about genetic causes of neurodevelopmental disorders, the more we are confronted with the fact that the same aetiology can lead to very variable outcomes. This is true for a wide range of genetic conditions that involve copy-number variants or point mutations, as well as chromosome aneuploidy ( Mitchell, 2015). Conditions such as tuberous sclerosis, 22q11.2 deletion syndrome, 15q11.2 microdeletion syndrome are well-established as risk factors for neurodevelopmental disorders, but the clinical manifestations can vary widely from person to person, even within a family. The phenotypic outcomes of these conditions do not respect traditional diagnostic boundaries: the same genetic aetiology can lead to autism, schizophrenia, epilepsy or ADHD. It is often assumed that this variability is due either to interactions with the genetic background or to environmental factors that moderate gene effects. This kind of explanation cannot, however, explain why in twin studies, one can often see marked phenotypic variability in concordant MZ twins: this has been shown by Le Couteur *et al.* (1996) for autism and by Corey *et al.* (2011) for epilepsy. Given that MZ twins have identical genes and closely similar environments, we need another explanation for phenotypic variability. One candidate is stochastic variation ( Molenaar *et al.*, 1993; Wolf, 1997). Purely random events early in neurodevelopment can have cascading effects so that quite minor differences in the prenatal brain lead to long-lasting differences in function. This alone, however, does not explain why there should be higher rates of impairment in those with chromosome trisomies. To account for that, we need to introduce the idea of developmental instability, i.e., a disturbance of the usual homeostatic processes that make the organisation robust to external influences ( Nijhout & Davidowitz, 2003). This would mean that small stochastic effects would have larger impact than would otherwise be the case.

Evidence that aneuploidy may be linked to developmental instability was obtained by Beach *et al.* (2017), who found high variability in cell cycle progression among yeast cells with identical aneuploidies. Furthermore, these cells showed variable response to environmental stress. These authors went on to demonstrate wide phenotypic variation in inbred mice with genetically engineered trisomy 19, despite a uniform genetic background and environment. Mitchell (2018) proposed that a wide range of genetically-based neurodevelopmental disorders may involve a process of developmental instability. This can make sense of the wide range of outcomes seen with an extra sex chromosome - both in terms of the severity of impairments, and in terms of the wide range in symptoms that are observed. If the main effect of an additional chromosome is to increase neurodevelopmental instability, then the developing brain will be less robust to perturbations, so that small differences in early neurodevelopmental processes will have cascading effects that can lead to very different clinical pictures.

### Clinical implications

When a sex chromosome trisomy is identified prenatally, it is important that parents are given accurate information about the implications for the child’s development; the quality of information provided is one factor that influences whether the mother continues with the pregnancy ( Jeon *et al.*, 2012). It is crucial that parents are provided with information from unbiased samples: The recommendations of Linden *et al.* (2002) are generally still valid: as they noted, children with sex chromosome trisomies ‘are at an increased risk for developmental problems but… most are in the normal range of development, and marked abnormality is not usually seen’ (p. 4). However, at the time they were writing, there was no awareness of the risk of autism, possibly because the diagnostic criteria for autism were far more stringent at the time when the neonatal screening studies were conducted ( Bishop *et al.*, 2011). The companion paper on this sample confirms the recommendations that parents should be informed that presence of a sex chromosome trisomy raises the probability that the child will have educational difficulties ( Bishop *et al.*, 2018), but the current analysis indicates that in addition, although the majority of children will not merit a clinical diagnosis for autism and/or Social Phobia, the likelihood of these conditions is increased.

Advice on management of children with a sex chromosome trisomy has mostly focused on the provision of speech and language therapy and educational interventions to address problems with speech, language and educational progress that may arise. The current study emphasises that symptoms of social anxiety are common in all three trisomies, suggesting that early involvement of Child and Adolescent Mental Health Services may be warranted in some cases. The outcomes of children with sex chromosome trisomies are very varied, making it difficult to predict in advance the exact type of support that will be needed, but it is clear that problems often do not fit neatly into single diagnostic categories, and optimal support may require collaboration between different services.

## Data availability

### Underlying data

Data, a data dictionary and R markdown scripts used for analyses are available from Open Science Framework

OSF: Dataset 1. Language and behavioural phenotypes in children with sex chromosome trisomies, https://doi.org/10.17605/OSF.IO/U2C7D ( Bishop, 2019)

License: CC0 1.0 Universal

### Reporting guidelines

OSF: STROBE checklist for “Autism and social anxiety in children with sex chromosome trisomies: an observational study”, https://doi.org/10.17605/OSF.IO/U2C7D ( Bishop, 2019)

License: CC0 1.0 Universal
