# Supplementary material for: Autism and social anxiety in children with sex chromosome trisomies: an observational study
Source: Wellcome Open Res. 2019 Sep 2;4:32. Originally published 2019 Feb 15. [Version 2] doi: 10.12688/wellcomeopenres.15095.2 (PMC6567293; doi:10.12688/wellcomeopenres.15095.2)
Supplement: Peer review history

# Peer review: Reviewer response for version 1

**DOI:** 10.21956/wellcomeopenres.16469.r35468

**Published:** 2019-06-03

**Authors:** Jobst Meyer

**Affiliations:** 1 Department of Neurobehavioral Genetics, Institute of Psychobiology, University of Trier, Trier, Germany

Wilson, King and Bishop present a well written manuscript focusing on features of autism and social phobia in three samples with trisomies of the sex chromosomes (XXX, XXY and XYY). They found elevated risks for autism-related symptoms in these groups.

 I have minor suggestions to improve this paper:

1. Abstract, background: The word “is” is missing somewhere in this sentence.
2. Abstract, results, third sentence: “…gave odds ratios (Plural)…”
3. Methods, participants, second-last sentence: “An unexpected feature (…) was that the majority of girls with trisomy X were in the Low Bias subgroup…” This observation is far from trivial – for example, with respect to genetic counseling – and better to be placed in the discussion. A recent paper by Freilinger and colleagues (2018) ^1^ might help to sort out whether the feature addressed here is really to be classified as “unexpected”.
4. Figure 2: Please specify the meaning of the solid and the dotted lines in the legend.
5. Figures 3 and 4: Since these two figures represent some kind of summaries, I would recommend to reword the legends in a way that these summaries are understandable even without the context of the paper; i.e., add some more information.
6. Discussion: Please be more concrete of how the information presented in this manuscript could help clinicians in the process of genetic counseling.

I confirm that I have read this submission and believe that I have an appropriate level of expertise to confirm that it is of an acceptable scientific standard.

## References

[1] : Behavioral and psychological features in girls and women with triple-X syndrome. *Am J Med Genet A*.2018;176(11) : 10.1002/ajmg.a.40477 2284-2291 10.1002/ajmg.a.40477 30070765

# Response

**Published:** 2019-08-24

**Authors:** Dorothy Bishop

**Affiliations:** University of Oxford, UK

Many thanks for your careful reading of this paper. We have made clarifications and corrections as you suggested, plus a further comment on the rates of impairment in females with trisomy X in the latest version of the paper.

# Peer review: Reviewer response for version 1

**DOI:** 10.21956/wellcomeopenres.16469.r35140

**Published:** 2019-04-08

**Authors:** Armin Raznahan

**Affiliations:** 1 Developmental Neurogenomics Unit (DNU), National Institute of Mental Health (NIMH) , Bethesda, MD, USA

This is an excellently designed, executed and reported study on autism and social anxiety in children with sex chromosome trisomies.

 I see no changes that would be required to improve upon the internal validity of this report.

 The authors provide a helpful and adequately referenced introduction, which precisely frames their primary hypotheses of interest.

 Study methods are presented in a detailed and transparent manner. Notable design strengths include consideration of multiple SCA groups and measurement types, as well as careful delineation and analysis of potential biases.

 The statistical analyses used are appropriate and clearly reported.

 The results are presented and discussed in a clear and nuanced manner - providing a balanced view of what can and can't be concluded from the work.

 One potential area where further discussion could help readers I think would be around the distinction between ASD and Social Anxiety Disorder. These presentations are very challenging to distinguish phenomenologically, and as such, online administration of the DAWBA may place limits on the accuracy of diagnostic classification vs. gold standard clinical interview for this particular diagnostic distinction. I also think that some discussion around the conceptual complexity  of "co-morbid" ASD and social anxiety disorder would be valuable for readers.

I confirm that I have read this submission and believe that I have an appropriate level of expertise to confirm that it is of an acceptable scientific standard.

# Response

**Published:** 2019-08-24

**Authors:** Dorothy Bishop

**Affiliations:** University of Oxford, UK

Many thanks for the comments on this paper.

The point about diagnostic difficulty is very pertinent, and a paragraph has been added to the latest version of the paper, giving a couple of illustrative vignettes.
